# The association of resistance training with risk of ovarian cancer

**DOI:** 10.1002/cam4.3804

**Published:** 2021-03-11

**Authors:** Andrea L Buras, Cassandra A Hathaway, Tianyi Wang, Mary K Townsend, Shelley S Tworoger

**Affiliations:** ^1^ Department of Gynecologic Oncology Moffitt Cancer Center Tampa FL USA; ^2^ Department of Obstetrics & Gynecology University of South Florida Tampa FL USA; ^3^ Department of Cancer Epidemiology Moffitt Cancer Center Tampa FL USA; ^4^ Department of Epidemiology Harvard T.H. Chan School of Public Health Boston MA USA

**Keywords:** cohort study, exercise, ovarian cancer, resistance training

## Abstract

**Background:**

Increasing evidence, including multiple putative inflammatory risk factors (e.g., c‐reactive protein, and adiposity), supports that inflammation plays an important role in ovarian carcinogenesis. Resistance training (RT) is associated with lower levels of circulating inflammatory markers, independent of physical activity.

**Methods:**

We evaluated the relationship between RT and risk of ovarian cancer accounting for other physical activity (e.g., walking) in two large prospective cohorts, the Nurses’ Health Study (NHS) and NHSII.

**Key Results:**

In total, analyses included 42,005 NHS participants (2000–2016) and 67,289 NHSII participants (2001–2017) with RT assessed every 4 years. Multivariable Cox proportional hazards models were used to estimate hazard ratios (HRs) and 95% confidence intervals (CIs) of RT with ovarian cancer risk overall and by tumor subtype, adjusting for known and putative ovarian cancer risk factors. We identified a total of 609 cases over 1,748,884 person‐years. No association was observed with overall ovarian cancer risk (RT ≥60 vs 0 min/wk, HR = 0.95, 95%CI: 0.74–1.22) or by histotype (comparable HR = 0.86 and 0.98 for type I and II tumors, respectively). Results did not differ by body mass index (Pinteraction = 0.97), or other physical activity (Pinteraction = 0.31).

**Conclusions & Inferences:**

We observed no evidence that moderate levels of RT were associated with risk of ovarian cancer. Further investigations are required to confirm these findings.

## Graphical abstract

Inflammation is thought to play a role in ovarian carcinogenesis. Physical activity and resistance training may reduce risk via lowering inflammation, although prior studies have had mixed results. In one of the largest prospective cohort studies evaluating resistance training and ovarian cancer risk, we observed no associations. Future work should explore the combination of modifiable anti‐inflammatory factors that decreases ovarian cancer risk.

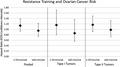

## 1 INTRODUCTION

Ovarian cancer is the fifth leading cause of cancer‐related death for women in the United States.^1^ The high mortality associated with ovarian cancer is related to its late stage of diagnosis with approximately 60% of women being diagnosed with distant spread of disease.^1^ Thus, consideration of whether lifestyle factors may influence the incidence of ovarian cancer may provide new opportunities for intervention.

Increasing evidence supports inflammation as having an important role in ovarian carcinogenesis.^2^, ^3^, ^4^ For example, inflammatory exposures, such as C‐reactive protein (CRP)^4^ and premenopausal obesity,^5^, ^6^, ^7^ are related to higher ovarian cancer risk. Alternatively, physical activity, which is associated with lower inflammation,^8^, ^9^ has been associated with decreased risk of several cancers,^10^ with inconsistent results for ovarian cancer risk.^10^, ^11^, ^12^ One aspect of activity that has received little attention is resistance training (RT), which is associated with reduced subclinical inflammation and lower levels of pro‐inflammatory markers in women, independent of physical activity.^8^, ^9^, ^13^ Thus, we evaluated the relationship of RT with ovarian cancer risk overall and by histology, as well as stratified by BMI and levels of other forms of physical activity (e.g., walking), utilizing data in two large prospective cohort studies, the Nurses’ Health Studies (NHS) and NHSII.

## 2 MATERIALS AND METHODS

### 2.1 Study population

NHS consists of 121,700 U.S. female registered nurses age 30–55 at the study's initiation in 1976.^14^ NHSII includes 116,429 U.S. female registered nurses, age 25–42 at enrollment in 1989.^15^ Participants from both cohorts completed a baseline questionnaire including questions on lifestyle, reproductive factors, and health history, as well as follow‐up questionnaires biennially to update this information as well as obtain information on cancer diagnoses. The study protocol was approved by the institutional review boards of the Brigham and Women's Hospital and Harvard T.H. Chan School of Public Health, and Moffitt Cancer Center (Advarra, PRO00022823), and those of participating registries as required. Consent was implied by return of questionnaires.

#### 2.1.1 Resistance training assessment

Questions regarding RT were asked every four years starting in 2000 (NHS) and 2001 (NHSII), with good reproducibility and validity.^16^ Participants were asked about average time spent weekly during the past year doing weight training or resistance exercises (include free weights or machines) for arm and leg weights separately. Answers were provided in categories of zero minutes, 1–4 min, 5–19 min, 20–59 min, 1 h, 1–1.5 h, 2–3 h, 4–6 h, 7–10 h, or 11+ hours per week. To estimate total RT time per week, each category was assigned the median value for that category, then values for arm and leg weights were summed. The total time RT per week was classified as no RT, 1–59 minutes, or 60+ minutes per week.

#### 2.1.2 Assessment of covariates

Data on weight, smoking, other physical activity, oral contraceptive use, parity, family history of breast or ovarian cancer, menopausal status, hormone therapy use, tubal ligation, and hysterectomy were regularly queried every 2 to 4 years. Physical activity, other than RT, was assessed on the same questionnaires as RT every four years, with the same categories as RT described above. Participants were asked about average time spent weekly during the past year walking, jogging, running, bicycling, swimming, playing tennis, or doing aerobic exercise, lower intensity exercise (yoga and stretching), and other vigorous activities (lawn mowing). For the analysis, each category of physical activity was assigned the median value for that category; then, a sum of all the physical activities was calculated to estimate continuous total time spent doing non‐RT physical activity per week.

#### 2.1.3 Ascertainment of incidence of ovarian cancer and death

Incident cases were identified by self‐report on the biennial questionnaires or linkage to the National Death Index,^17^, ^18^ and were confirmed through review of medical records, including pathology reports, or linkage to the relevant cancer registry. A gynecologic pathologist, blinded to exposure status, reviewed pathology reports to abstract morphology, stage, histology, grade, and invasiveness of the tumor. A sample of 417 ovarian cancer cases previously compared concordances of the pathologist's review of slides and pathology reports for carcinoma versus borderline, grade, and histologic type and found concordance of 94%, 79%, and 78%, respectively.^19^

### 2.2 Statistical methods

Women with a bilateral oophorectomy, history of cancer other than non‐melanoma skin cancer, missing date of birth or death, or menopause due to radiation were excluded at baseline. Cox proportional hazards model was used to estimate hazard ratios (HRs) and 95% confidence intervals (CI). Model 1 was stratified on age, calendar year, and cohort, with adjustment for body mass index (BMI; continuous), oral contraceptive use (never, <1 year, 1‐<5 years, 5‐<10 years, 10+ years, and unknown), parity (nulliparous, 1 child, 2 children, 3 children, and 4 children), family history of breast or ovarian cancer (yes and no), menopausal status (premenopausal, postmenopausal, and unknown), smoking status (never, past, and current), hormone therapy use (estrogen, estrogen, and progesterone, other hormone therapy use: never, ever), history of tubal ligation (yes and no), and history of hysterectomy (yes and no). Model 2 additionally adjusted for cumulatively averaged minutes of physical activity per week (continuously; excluding RT). *p*‐values for trend per 10 min of RT (continuous) were calculated.

We assessed whether associations differed by tumor histotype [Type I (low‐grade serous, endometrioid, clear cell, or mucinous) versus Type II tumors (high‐grade serous, poorly differentiated or transitional/Brenner)] using competing risks Cox models and tested potential heterogeneity in effect estimates using a likelihood ratio test comparing models holding the association across histotypes constant versus allowing them to vary. To test for differences between mucinous and other Type I tumors, we conducted a sensitivity analysis limited to low‐grade serous, endometrioid, and clear cell tumors.

In stratified analyses, we assessed risk of ovarian cancer with cumulatively averaged RT by BMI (<25, ≥25 kg/m^2^) and other physical activity (below or above the 150 minutes per week^20^). *p*‐values for interaction were calculated using a likelihood ratio test comparing models with versus without interaction terms. To test for heterogeneity by cohort, HRs were calculated separately in each cohort and pooled using random‐effects meta‐analysis. We also cross‐classified RT with other physical activity (no RT and <150 min/week of physical activity, any amount of RT and <150 min/week of physical activity, no RT and ≥150 min/week of physical activity, and any amount of RT and ≥150 min/week of physical activity), and BMI (no RT and BMI <25 kg/m^2^, any amount of RT and BMI <25 kg/m^2^, no RT and BMI ≥25 kg/m^2^, and any amount of RT and BMI ≥25 kg/m^2^), and evaluated their associations with ovarian cancer risk.

Additional analyses included using the most recent RT measurement before ovarian cancer diagnosis instead of cumulative average as the exposure, as well as cumulative average RT during the premenopausal period only; these latter analyses were conducted because our previous study of total physical activity only observed an association with premenopausal exercise.^21^ All statistical tests were two‐sided and conducted with SAS, version 9.4 (SAS Institute Inc., Cary, North Carolina, United States).

## 3 RESULTS

A total of 109,294 women (1,748,884 person‐years), including 609 cases of ovarian cancer, were included in the analysis. At first assessment of RT, the mean age was 65.8 years in NHS and 46.4 years in NHSII. Those with more minutes of RT had lower BMI, higher amounts of other physical activity, were more likely to be nulliparous or a past smoker, and less likely to be current smokers. (Table 1).

**TABLE 1:** Age‐standardized characteristics of the study population in NHS (2000) and NHSII (2001) by resistance training time at baseline.

	NHS (*n* = 42,005)			NHSII (*n* = 67,289)		
	0 min/week (*n* = 32,255)	1–59 min/week (*n* = 4949)	≥60 min/week (*n* = 4801)	0 min/week (*n* = 43,674)	1–59 min/week (*n* = 10,460)	≥60 min/week (*n* = 13,155)
Age, years, mean (sd)	66.1 (7.2)	65.4 (7.1)	64.1 (6.6)	46.5 (4.6)	46.2 (4.6)	46.1 (4.6)
BMI, mean (sd)	27.3 (5.6)	25.8 (4.8)	25.2 (4.5)	27.5 (6.6)	25.5 (5.2)	24.9 (4.6)
Physical activity minutes per week (excluding resistance training), mean (sd)	21.9 (26.5)	31.1 (28.3)	48.6 (38.5)	23.5 (29.3)	32.3 (28.5)	57.1 (46.3)
Oral contraceptive use, %						
Never	48.6	47.0	45.2	14.2	13.3	12.1
<1 year	12.9	13.4	13.3	8.2	8.5	8.0
1–<5 years	21.1	22.2	22.6	35.5	36.9	35.5
5–<10 years	12.1	12.3	12.8	25.0	24.5	25.2
10+ years	5.1	4.9	6.0	15.0	14.7	17.2
Unknown	0.2	0.2	0.1	2.0	2.2	2.0
Parity, %						
Nulliparous	4.8	4.6	5.5	17.4	16.7	20.0
1 child	6.8	6.6	6.1	14.1	12.8	13.2
2 children	27.6	27.7	29.2	39.4	40.9	39.3
3 children	29.1	29.9	29.8	20.9	22.2	20.6
≥4 children	31.6	31.1	29.4	8.1	7.4	6.9
Family history of breast or ovarian cancer, %	19.3	19.6	19.0	13.6	13.7	14.0
Smoking, %						
Never smoked	45.0	44.4	41.7	67.0	66.9	63.9
Past smoker	43.7	50.1	53.3	23.5	26.9	29.8
Current smoker	11.3	5.5	5.0	9.5	6.2	6.3
Postmenopausal, %	97.2	97.0	97.1	14.7	14.4	14.9
Ever estrogen use, %^a^	25.9	28.7	30.7	4.4	5.8	5.0
Ever estrogen & progesterone Use, %^a^	35.7	42.3	46.2	48.9	54.2	55.9
Ever other hormone therapy use, %^1^	22.4	26.5	29.5	7.8	8.6	9.0
Tubal ligation, %	21.5	20.5	22.2	26.7	24.8	24.5
Hysterectomy, %	22.7	22.9	23.1	9.1	7.9	8.4

Abbreviations: BMI, body mass index; MET, metabolic equivalent of task; NHS, Nurses’ Health Study; SD, standard deviation.

^a^ Among postmenopausal women.

As no heterogeneity was found across cohorts (*p*‐heterogeneity =0.51; Table S1), data from NHS and NHSII were pooled for all further analyses. No association with ovarian cancer risk was observed for RT (Table 2, Model 2; ≥60 min/week vs. no RT, HR =0.95, 95% CI: 0.74–1.22; *p*‐trend =0.26). There was no observed heterogeneity by histotype (comparable HR=0.86 and 0.98 for type I and II tumor, respectively; p‐heterogeneity=0.79). We observed no differences between all type I tumors and a sensitivity analysis excluding mucinous tumors (data not shown).

**TABLE 2:** Association of cumulative average resistance training with ovarian cancer risk overall and by tumor histotype in the NHS and NHSII.

Cumulative average resistance training, HR (95% CI)				
	0 min/week	1–59 min/week	≥60 min/week	*p*‐trend^a^
Pooled				
Cases/person‐years	364/981,615	150/432,930	95/334,339	
Model 1	ref	1.15 (0.94, 1.40)	0.98 (0.78, 1.24)	0.43
Model 2	ref	1.14 (0.93, 1.39)	0.95 (0.74, 1.22)	0.26
Type I Tumors^b^, ^c^				
Cases/person‐years	92/981,321	41/432,807	26/334,266	
Model 1	ref	1.18 (0.81, 1.73)	0.91 (0.58, 1.42)	0.45
Model 2	ref	1.16 (0.80, 1.71)	0.86 (0.55, 1.36)	0.29
Type II Tumors^c^, ^d^				
Cases/person‐years	247/981,492	103/432,885	65/334,310	
Model 1	ref	1.19 (0.93, 1.51)	1.04 (0.78, 1.38)	0.47
Model 2	ref	1.17 (0.92, 1.49)	0.98 (0.73,1.32)	0.24

Model 1: HRs were calculated using Cox proportional hazards models stratified by age (continuous), calendar year (continuous), and cohort (NHS and NHSII), and adjusted for BMI (continuous), oral contraceptive use (never, <1 yr, 1‐<5 yrs, 5‐<10 years, 10+yrs, and unknown), parity (nulliparous, 1 child, 2 children, 3 children, and 4 children), family history of breast or ovarian cancer (yes and no), menopausal status (premenopausal, postmenopausal, and unknown), smoking (never, past, and current), hormone therapy use (estrogen, estrogen+progesterone, and other hormone therapy use: never, ever), tubal ligation (yes and no), and hysterectomy (yes and no).

Model 2: Model 1 plus further adjustment for other physical activity (continuous, cumulatively averaged minutes per week of walking, running, jogging, biking, swimming, tennis, aerobics, yoga, and lawn work).

Abbreviations: CI, Confidence Interval; HR, Hazard Ratio; NHS, Nurses’ Health Study.

^a^ Per 10 min of resistance training.

^b^ Type I Tumors = Low‐grade serous, endometrioid, clear cell, and mucinous.

^c^ *p*‐heterogeneity comparing Type I and Type II Tumors for Model 1 and 2 = 0.79.

^d^ Type II Tumors =High‐grade serous, Transitional/Brenner

No association was observed by strata of other physical activity (*p*‐interaction =0.31) or BMI (*p*‐interaction =0.97; Table 3). Further, no clear association was found with most recently reported RT, premenopausal cumulative average RT, or RT cross‐classified with physical activity or BMI (data not shown).

**TABLE 3:** Association of cumulative average resistance training with ovarian cancer risk overall and stratified by other physical activity and BMI in the NHS and NHSI.

Cumulative average resistance training				
	0 min/week	1–59 min/week	≥60 min/week	*p*‐trend^a^
Other Physical Activity				
<150 min/week				
Cases/person‐years	330/897,523	125/350,505	64/212,889	
HR (95% CI)^b^	Ref	1.16 (0.93, 1.44)	0.97 (0.73, 1.29)	0.48
≥ 150 min/week				
Cases/Person‐years	34/84,092	25/82,425	31/121,450	
HR (95% CI)^b^	ref	0.99 (0.57, 1.71)	0.83 (0.49, 1.42)	0.34
BMI				
<25 kg/m^2^				
Cases/person‐years	130/331,450	60/187,592	49/162,276	
HR (95% CI)^c^	ref	1.07 (0.78, 1.49)	1.03 (0.71, 1.47)	0.27
≥25 kg/m^2^				
Cases/person‐years	234/650,166	90/245,338	46/172,063	
HR (95% CI)^c^	ref	1.22 (0.95, 1.58)	0.89 (0.63, 1.26)	0.60

HRs were calculated using Cox proportional hazards models stratified by age (continuous), calendar year (continuous), and cohort (NHS, NHSII), and adjusted for BMI (continuous), oral contraceptive use (never, <1 yr, 1‐<5 yrs, 5‐<10 yrs, 10+yrs, and unknown), parity (nulliparous, 1 child, 2 children, 3 children, and 4 children), family history of breast or ovarian cancer (yes and no), menopause status (pre, post, and unknown), smoking (never, past, and current), hormone therapy use (estrogen, estrogen+progesterone, and other hormone therapy use: never, ever), tubal ligation (yes and no), hysterectomy (yes and no) and other physical activity (continuous, cumulatively averaged minutes per week of walking, running, jogging, biking, swimming, tennis, aerobics, yoga, and lawn work).

Abbreviations: CI, confidence interval; HR, hazard ratio; NHS, Nurses’ Health Study.

^a^ per 10 min of resistance training.

^b^ *p*‐interaction for other physical activity =0.31.

^c^ *p*‐interaction for BMI =0.97.

## 4 DISCUSSION

This large longitudinal study assessing the relationship between RT and risk of ovarian cancer accounting for other physical activity observed no association of RT with ovarian cancer risk overall or by histotype in two large prospective cohort studies, though due to limited power in histotypes, we cannot rule out a modest association in individual tumor types. Further, we did not observe any associations when stratifying by adiposity or other physical activity.

To our knowledge, no other studies have assessed the relationship between RT and ovarian cancer risk specifically; however, studies have evaluated RT with risk of other cancers. In a case–control study of 870 cases and 996 controls in Australia, no association of RT was found with risk of colon or rectal cancer.^22^ However, in a cohort study of members of the American Association of Retired Persons in the United States, compared to no weightlifting, weightlifting 5 minutes to 1.5 hours per week and weightlifting 2 to 10+ hours per week were associated with significant 22% and 25% reduced risks of colon cancer, respectively, although these associations were observed only in males.^23^ In a large prospective cohort study of men, RT was not associated with total cancer risk, though marginal reduced risks of bladder and kidney cancer were observed.^24^ While more research is needed, it appears that RT may not be strongly related to cancer risk, despite a randomized control trial of RT showing a decreased risk of metabolic syndrome and improved inflammatory markers.^8^ In observational studies, RT also has been related to decreased risk of metabolic syndrome^8^ and lower inflammatory markers, particularly CRP^2^, ^8^, ^13^ in observational studies. Overall, additional larger studies are needed to assess this association with ovarian cancer to determine with a modest association exists.

Ovarian cancer is a complex disease with varying risk factors by tumor histology.^25^ For example, higher BMI and endometriosis increase the risk of type I tumors such as mucinous, endometroid, and clear cell, more so than type II tumors such as serous.^6^, ^25^ Additionally, higher levels of CRP were more strongly positively associated with mucinous and endometrioid tumors, than serous disease^4^ indicating that type I tumors may be susceptible to effects of chronic inflammation. Although we did not see an association between RT and ovarian cancer risk in type I or type II tumors, our power was limited and we were unable to look at individual histological subtypes. Larger studies which pool multiple cohorts are warranted to understand the association of RT with specific histological subtypes.

Our study is limited to self‐reported physical activity and RT, which may cause misclassification in the exposure assessment, although validation of the questionnaire with physical activity diaries showed good correlations.^16^ Frequency of RT was not accessed, precluding examination of associations with meeting specific recommended activity guidelines (i.e., 150 min of physical activity and RT twice a week).^20^ Additionally, while the cohort size overall is large, we had limited power to assess the association by histotype, and because most women were postmenopausal at the first RT assessment, few women had assessment of RT during the premenopausal period. Our sample also consisted of mostly white female nurses. Nevertheless, this study had a number of strengths, including the prospective design, repeated exposure assessments, and long‐term follow‐up, with detailed information on potential confounders.

In summary, our data did not demonstrate evidence that RT was associated with risk of ovarian cancer. Modifiable risk factors that decrease inflammation such as RT likely each play a small role, making it difficult to identify associations between individual factors and ovarian cancer risk. Future work should focus on considering multiple lifestyle factors in combination with ovarian cancer risk and assessing circulating inflammatory markers and their relationship to RT, which may provide insight into biological mechanisms that may influence ovarian cancer risk.

## 5 ETHICS STATEMENT

The study protocol was approved by the institutional review boards of the Brigham and Women's Hospital and Harvard T.H. Chan School of Public Health, and Moffitt Cancer Center (Advarra, PRO00022823), and those of participating registries as required. Consent was implied by return of questionnaires.

## CONFLICTS OF INTEREST

The authors declare no potential conflicts of interest.

## AUTHOR CONTRIBUTION

All of the authors made substantial contributions to the study concept, design, analysis, and interpretation of the data. Specifically, Andrea L Buras was involved in interpretation of the results and the drafting of the manuscript. Cassandra A Hathaway contributions include designing of the study's analytic strategy, conducting the analysis, analyzing the results, and drafting the manuscript. Tianyi Wang contributed to the study's analytic strategy, interpretation of the results, double checking of the final results, and contributed to manuscript revisions. Mary K Townsend contributed to analyzing the data, the study's analytic strategy, and manuscript revisions. Shelley S Tworoger conceived of and directed the studies and contributed to manuscript revision.

## Supporting information

Table S1

## Data Availability

Data available on request from the authors.
