# Supplementary material for: The association of resistance training with risk of ovarian cancer
Source: Cancer Med. 2021 Mar 11;10(7):2489–95. doi: 10.1002/cam4.3804 (PMC7982607; doi:10.1002/cam4.3804)
Supplement: Supplementary file 1 — Table S1 [file CAM4-10-2489-s001.docx]

| **Supplemental Table 1.** Stratified analyses of cumulative averaged resistance training and ovarian cancer risk by cohort | | | | |
| --- | --- | --- | --- | --- |
| **Cumulative average resistance training** | | | | |
|  | 0 min/wk | 1-59 min/wk | ≥60 min/wk | p-trend^†^ |
| **Nurses' Health Study** | | | | |
| Cases/Person-years | 244/419,371 | 89/141,111 | 51/91,030 |  |
| HR (95% CI)^‡^ | ref | 1.18 (0.92, 1.52) | 1.01 (0.73, 1.40) | 0.66 |
| **Nurses' Health Study II** | | | | |
| Cases/Person-years | 120/562,244 | 61/291,819 | 44/243,309 |  |
| HR (95% CI)^‡^ | ref | 1.06 (0.77, 1.46) | 0.87 (0.60, 1.27) | 0.23 |

Abbreviations: CI, confidence interval; HR, hazard ratio

HRs were calculated using Cox proportional hazards models stratified by age (continuous), calendar year and cohort, and adjusted for BMI (continuous), oral contraceptive use (never, <1yr, 1-<5yrs, 5-<10yrs, 10+yrs), parity (nulliparous, 1 child, 2 children, 3 children, 4 children), family history of breast or ovarian cancer (yes, no), menopause status (pre, post, unknown), smoking (never, past, current), hormone therapy use (estrogen, estrogen+progesterone, other hormone therapy use: never, ever), tubal ligation (yes, no), hysterectomy (yes, no) and other physical activity (continuous, cumulatively averaged minutes per week of walking, running, jogging, biking, swimming, tennis, aerobics, yoga, lawn work)

^†^per 10 minutes of resistance training

^‡^p-heterogeneity between cohorts = 0.51
